# Effectiveness and Safety of Lenvatinib and Everolimus after Immune Checkpoint Inhibitors in Metastatic Renal Cell Cancer: A Systematic Review

**DOI:** 10.32604/or.2025.070523

**Published:** 2025-12-30

**Authors:** Giacomo Iovane, Luca Traman, Michele Maffezzoli, Giuseppe Fornarini, Domenico Corradi, Debora Guareschi, Matteo Santoni, Sebastiano Buti

**Affiliations:** 1 Medical Oncology Unit, University Hospital of Parma, Parma, 43126, Italy; 2 Medical Oncology Unit, University Hospital of Genova, Genova, 16132, Italy; 3 Medical Oncology Unit, Portsmouth Hospitals University NHS Trust, Portsmouth, PO6 3LY, UK; 4 Pathology Unit, University Hospital of Parma, Parma, 43126, Italy; 5 Medical Oncology Unit, Hospital of Macerata, Macerata, 62100, Italy

**Keywords:** Metastatic renal cell carcinoma (mRCC), immune checkpoint inhibitors (ICIs), lenvatinib, everolimus, effectiveness, safety, systematic review

## Abstract

**Background:**

While the treatment of metastatic renal cell carcinoma (mRCC) is evolving due to immune checkpoint inhibitors (ICIs), optimal strategies for later lines of therapy have yet to be defined. The combination of lenvatinib and everolimus represents a viable option, and the present review aimed to summarize its activity, effectiveness, and safety.

**Methods:**

A systematic review of the literature was conducted using PubMed, targeting studies published between 2018 and 2025. Eligible studies included English-language prospective and retrospective trials reporting survival outcomes in mRCC patients treated with lenvatinib and everolimus after at least one ICI-containing regimen.

**Results:**

Nine studies met the inclusion criteria, encompassing a total of 441 patients. The lenvatinib and everolimus combination was primarily used in the third and subsequent lines of therapy. Median overall survival ranged from 7.5 to 24.5 months, while median progression-free survival was more consistent, between 6.1 and 6.7 months, except for one study reporting 12.9 months. Objective response rates varied widely (14.0%–55.7%). Adverse events of grade ≥ 3 did not exceed the expected rate, with diarrhoea and proteinuria as the most reported events. Dose reductions and treatment discontinuations due to toxicity occurred but were generally lower than in prior pivotal trials.

**Conclusions:**

Real-world evidence suggests that lenvatinib and everolimus represent an effective and safe option after ICI failure in mRCC patients. Nevertheless, the lack of randomized phase III trials and the heterogeneity of existing studies highlight the need for more robust prospective research to guide post-ICI therapeutic strategies.

## 1 Background

Kidney cancer represents the 14th most common malignancy worldwide, with more than 430,000 cases diagnosed in 2020. Epidemiological data show a heterogeneous geographical distribution of this disease, with higher incidence in Europe and North America [1]. Even though 66% of renal cell cancers present as localized disease at diagnosis and show a 5-year relative survival of 93%, almost 15% of patients are diagnosed with metastatic disease, which is associated with a 5-year relative survival of less than 20% [2].

Over the last 7 years, the first-line treatment landscape for metastatic renal cell carcinoma (mRCC) has radically changed following the results of several phase III trials that showed the superiority of immune checkpoint inhibitors (ICIs) anti-programmed cell death protein 1 (PD-1) in combination either with a vascular endothelial growth factor receptor tyrosine kinase inhibitor (VEGFR-TKI) [3–5] or with a cytotoxic T-lymphocyte-associated protein 4 (CTLA-4) inhibitor [6] over VEGFR-TKI monotherapy, resulting in an increased median overall survival (mOS) of over 4 years. Thus, combination therapies (ipilimumab-nivolumab, lenvatinib–pembrolizumab, axitinib–pembrolizumab and cabozantinib–nivolumab) have become the recommended first-line option in both European Society of Medical Oncology (ESMO) and National Comprehensive Cancer Network (NCCN) guidelines [7,8].

Despite this marked improvement in the first-line setting, between 32.0% and 51.3% of patients will receive at least one further therapy, making the strategy of sequencing treatments a crucial point in the management of patients with mRCC [9,10]. Nevertheless, treatment strategies for second- and later lines are still not supported by high-quality evidence.

The combination of everolimus (a mammalian target of rapamycin (mTOR) inhibitor) and lenvatinib (a multitarget-TKI) represents one of the pursuable options as second- or subsequent lines of therapy and many studies have described its safety and efficacy in improving survival outcomes after progression on a first-line VEGFR-TKI monotherapy [11,12].

Many preclinical data support the synergic antineoplastic effect exerted by these two molecules. Lenvatinib targets multiple tyrosine kinase receptors, including VEGFR-1/2/3, platelet-derived growth factor receptor-β (PDGFR-β), rearranged during transfection (RET), c-KIT, and fibroblast growth factor receptors (FGFR) 1–4, which are involved in tumour growth and neoplastic angiogenesis mediated by constitutively activated hypoxia-inducible factors (HIF). Everolimus inhibits the mTOR pathway, responsible for cell metabolism, proliferation, apoptosis escape and metastatic dissemination. Moreover, hypoxia-inducible factor (HIF)-induced overexpression of growth factors (like VEGF) leads to mTOR activation in both renal and endothelial cells, which in turn enhances HIF expression, resulting in positive feedback between HIF- and mTOR pathways [13,14]. Several studies have also investigated the activity and effectiveness of lenvatinib and everolimus combinations after progression to a previous ICI-based regimen.

The aim of the present review is to describe the state of the art about the effectiveness and safety of the lenvatinib and everolimus combination in patients pretreated with ICIs.

## 2 Methods

### 2.1 Searching Strategy

This systematic review was conducted according to the Preferred Reporting Items for Systematic Reviews and Meta-Analyses (PRISMA) guidelines [15] and according to the Population Intervention Comparison Outcomes (PICO) [16] process (see Supplemental Materials for PICO criteria and PRISMA checklist). Two independent reviewers conducted a systematic review of publications involving patients with mRCC treated with the lenvatinib and everolimus combination, after having received at least one line of ICI-based therapy. Eligible studies were selected on the electronic database PubMed by using the following combination of MeSH terms: (“second-line” OR “third-line” OR “further line” OR “subsequent”) AND (“renal cell carcinoma” OR “kidney cancer” OR “renal cancer”) AND (metastatic OR advanced) AND (treatment OR therapy) NOT review NOT meta-analysis NOT “local therapy” NOT “local treatment” NOT guidelines.

In this MeSH search, only the articles published between 2018 and 2025 were included.

In order to ensure completeness, references from reviewed articles and preclinical studies were hand-searched. The final literature search was conducted on 03 September 2025.

### 2.2 Trial Selection Criteria

Our review included only publications in English. The inclusion criteria for study selection were as follows: clinical trials and retrospective or prospective observational studies involving patients with histologically confirmed mRCC; an intervention arm including both lenvatinib and everolimus; prior exposure of the study population to at least one line of ICI-based therapy; studies were required to report data on efficacy, effectiveness, activity and safety outcomes (the latter, when available).

Exclusion criteria included systematic reviews, meta-analyses, case-control studies, editorials, and commentaries. Additionally, studies from which relevant outcome data could not be extracted—despite meeting all other inclusion criteria—were excluded.

### 2.3 Outcomes Measurement

In order to evaluate the clinical outcomes of the treatments considered in our review, we analysed mOS, median progression-free survival (mPFS), and objective response rate (ORR). When reported in the selected studies, we considered median time to treatment failure (mTTF).

We also gathered data regarding adverse events (AEs) and their severity grading (from G1 to G4) according to Common Terminology Criteria for Adverse Events (CTCAE, version 5.0).

Finally, we defined the evidence quality of each study according to the GRADE system’s criteria (see Supplemental Materials for GRADE criteria) [17].

## 3 Results

### 3.1 Literature Search Results

**Figure 1:**
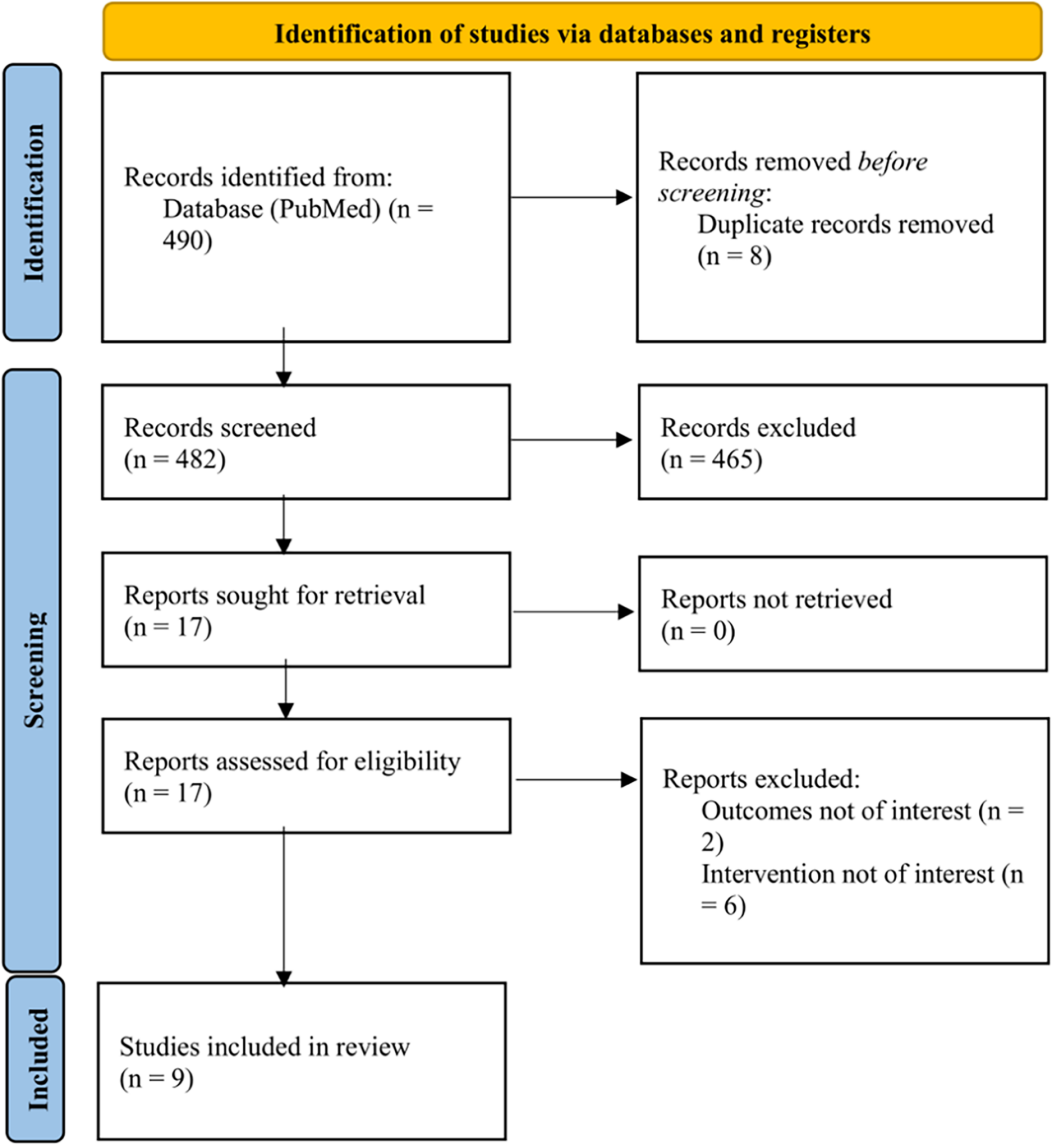
PRISMA flow diagram of the study selection process for systematic review. PRISMA, Preferred Reporting Items for Systematic Review and Meta-Analyses

A total of 490 potentially eligible studies were obtained by utilising the research criteria described above. After a first assessment of titles and abstracts, 473 articles were excluded due to irrelevance. The remaining 17 articles were full-text reviewed, which led to the further exclusion of eight publications. Finally, nine studies were included in our review (Fig. 1).

### 3.2 Trials Design and Main Characteristics

Most of the included studies were observational: seven were retrospective, mono- or multicentric studies, while the work by Hamieh et al. reported a case series [18]. The work by Pal et al. reported a prospective, randomized phase II trial [19]. All these works were published between 2020 and 2025 [17–25].

The International Metastatic RCC Database Consortium (IMDC) intermediate-risk group was the most consistently represented across all trials, ranging from 46.0% to 76.4% of the study populations, while the distribution of patients in the poor- and good-risk groups was more variable [18–26]. In eight studies, the effectiveness of lenvatinib and everolimus was evaluated from the second-line setting [18–22,24–26], while in the study by Wiele et al., the combination was administered from the third-line setting onwards [23]. Globally, the lenvatinib and everolimus combination was mainly used in the third- and further lines (from 6.3% to 43.0% and from 1.2% to 80.8%, respectively); in the work by Wiele et al. and in the work by Lee et al., nine patients (16.4%) and eight patients (9.6%) respectively received the treatment even as seventh- or eighth-line regimen [23,26].

The most represented evidence quality level according to GRADE criteria was “low”, since the majority of the included studies were observational; the work by Pal et al. was associated with a “high” level, being a randomized clinical trial [17,19].

Relevant results of all included articles were summarized in Table 1.

**Table 1:** Main characteristics of included studies and survival outcomes

Study reference, (year)	Study type	Evidence quality (GRADE)	Number of patients/total	Treatment line, n (%)	IMDC distribution, n (%)	ORR, *n* (%)	mPFS or PFS range (months)	mOS or OS range (months)	mTTF (months)
Hamieh et al. (2020) [18]	Case series	Very low	5 (out of 7)	2nd: 4 (57.0%)	“intermediate”: 4 (57.0%)	Not reported	3.0–9.0	4.0–11.0	
				3rd: 3 (43.0%)	“poor”: 3 (43.0%)				
Ged et al. (2020) [20]	Retrospective	Low	4 (out of 59)	2nd: 42 (71.0%)	“good”: 13 (22.0%)	25.00%	12.0 (95% CI, 8.2–24.5)**	24.5 (95% CI, 12.0–NE)	
				3rd: 17 (29.0%)	“intermediate”: 35 (59.0%)				
					“poor”: 11 (19.0%)				
Wiele et al. (2021) [23]	Retrospective	Low	42 (out of 55)	3rd: 4 (7.3%)	“good”: 6 (10.9%)	12 (21.8%)	6.2 (95% CI 4.8–9.4)	12.1 (95% CI 4.3–NA)	
				4th: 22 (40.0%)	“intermediate”: 42 (76.4%)				
				5th: 11 (20.0%)	“poor”: 7 (12.7%)				
				6th: 9 (16.4%)					
				≥7th: 9 (16.4%)					
Vogelzang et al. (2021) [22]	Retrospective	Low	79	2nd: 5 (6.3%)	“good”: 15 (19.0%)	34 (55.7%)	6.1 (95% CI 4.4–9.0)	14.8 (95% CI 10.2–23.9)	
				3rd: 18 (22.8%)	“intermediate”: 42 (53.2%)				
				4th: 25 (31.6%)	“poor”: 10 (12.7%)				
				5th: 16 (20.3%)	unknown: 12 (15.1%)				
				≥6th: 15 (19.0%)					
Kwok et al. (2023) [21]	Retrospective	Low	71	2nd: 2 (2.8%)	“good”: 14 (19.7%)	26.30%		9 (95% CI, 7.6–12.9)	5.5 (95% CI, 3.5–7.6)
				3rd: 19 (26.8%)	“intermediate”: 47 (66.2%)				
				4th: 15 (21.1%)	“poor”: 8 (11.3%)				
				Further lines: 35 (49.3%)	unknown: 2 (2.8%)				
Pal et al. (2022)* [19]	Randomized, phase II trial	High	90 (out of 343)	2nd: 129 (75.0%); 140 (82.0%)	“good”: 25 (15.0%), 38 (22.0%)	30.0% (95% CI 17–46), 51% (95% CI 35–68)	12.0,12.9 (90% CI, 0.74–2.20)	17.1, 18.0 (90% CI, 0.75–2.18)	
				3rd: 38 (22.0%); 29 (17.0%)	“intermediate”: 107 (62.0%),78 (46.0%)				
				≥4th: 5 (2.9%); 2 (1.2%)	“poor”: 40 (23.0%), 52 (30.0%)				
					unknown: 0 (0), 3 (1.8)				
Buti et al. (2024) [25]	Retrospective	Low	33	2nd: 2 (6.0%)	“good”: 4 (12.0%)	14 (42.0%)	6.7 (95% CI 4.9–not reached)	11.2 (95% CI 6.8–19.9)	
				3rd: 10 (30.0%)	“intermediate”: 19 (58.0%)				
				4th: 14 (42.0%)	“poor”: 10 (30.0%)				
				5th: 6 (18.0%)					
				6th: 1 (3.0%)					
Gavira et al. (2025) [24]	Retrospective	Low	45 (out of 133)	2nd: 10 (7.5%)	“good”: 21 (15.8%)	6 (14.0%)		9.6***	6.2***
				3rd: 49 (36.8%)	“intermediate”: 69 (51.9%)				
				4th and further: 74 (55.6%)	“poor”: 37 (27.8%)				
					unknown: 6 (5.0%)				
Lee et al. (2025) [26]	Retrospective	Low	72 (out of 87)	2nd: 7 (8.4%)	“good”: 6 (7.2%)	35.70%	5.3 (95% CI 4.3–7.4)	7.5 (95% CI 6.1–11.7)	
				3rd: 9 (10.8%)	“intermediate”: 58 (69.9%)				
				4th: 22 (26.5%)	“poor”: 19 (22.9%)				
				5th and further: 45 (54.3%)					

Note: *All results from the work by Pal et al. are separately reported for the population receiving Lenvatinib at 14 mg dose and the one receiving 18 mg dose; **In the work by Ged et al., the authors mentioned significant variability between IMDC groups in terms of mPFS and mOS but did not report separate results; ***These results describe mOS and mTTF in the global population, since separated results for patients who had previously received ICIs were not reported; IMDC, International Metastatic RCC Database Consortium; ORR, Objective Response Rate; mPFS, median Progression Free Survival; mOS, median Overall Survival; mTTF, median Time To Treatment Failure; CI, Confidence Interval; NE, Not Estimable; NA, Not Applicable.

### 3.3 Patient Populations

Globally, a total of 441 patients were treated with lenvatinib and everolimus combination. Across the trials, patients’ characteristics were quite similar.

The vast majority of patients were male (from 70.9% to 100%), with a median age at lenvatinib and everolimus initiation of 61 years (with a range from 22 to 87 years). Moreover, these patients were in good clinical condition at the beginning of the treatment, with a prevalence of Eastern Cooperative Oncology Group performance status (ECOG PS) of 0 or 1 (from 11.4% to 73.0% and from 26.0% to 67.1%, respectively). Only 110 patients (ranging from 16.5% to 35.4%) presented an ECOG PS ≥2; of note, only 6 studies reported ECOG PS values [19,22–26].

The most represented histology was clear cell carcinoma (from 64.0% to 100%); among the other histologies, the most common were the papillary (from 3.6% to 18.0%) and the chromophobe (from 1.3% to 7.3%). In the study by Pal et al., no data on histology were reported [19].

The lungs were the most common site for distant metastases (from 49.3% to 85.7%), followed by distant lymph nodes (from 25.3% to 78.2%). Brain (from 1.4% to 42.8%), bones (from 25.3% to 69.1%) and liver (from 11.3% to 43.6%) were also reported as frequent metastatic sites. In the study by Ged et al., metastatic sites were not reported [20].

All detailed patients’ characteristics are summarized in Table 2.

**Table 2:** Patients’ characteristics

Study reference, (year)	Sex, n (%)	Median Age, years (range)	ECOG PS, n (%)	Histology, n (%), [sarcomatoid features]	Metastatic sites, n (%)
Hamieh et al. (2020) [18]	Male: 7 (100%)	57 (39–63)	Not reported	Clear cell: 7 (100%) [3 (43.0%)]	Lung: 6 (85.7%)
					Lymph node: 4 (57.1%)
					Liver: 1 (14.2%)
					Brain: 3 (42.8%)
					Adrenal gland: 1 (14.2%)
					Bone: 4 (57.1%)
					Other: 1 (14.2%)
Ged et al. (2020) [20]	Male: 43 (78.0%) Female: 12 (22.0%)	55 (33–75)	Not reported	Clear cell: 55 (100%)	Not reported
Wiele et al. (2021) [23]	Male: 39 (70.9%) Female: 16 (29.1%)	62 (34–87)	0: 15 (27.3%) 1: 23 (41.8%) 2: 16 (29.1%) 3: 1 (1.8%)	Clear cell: 45 (81.8%) Papillary (1/2): 2 (3.6%) Chromophobe: 4 (7.3%) Translocation: 1 (1.8%) Unclassified: 3 (5.5%) [8 (14.5%)]	Lung: 46 (83.6%) Lymph node: 43 (78.2%) Liver: 24 (43.6%) Brain: 12 (21.8%) Bone: 38 (69.1%) Other: 26 (47.3%)
Vogelzang et al. (2021) [22]	Male: 58 (73.4%) Female: 21 (26.6%)	65 (33–85)	0: 9 (11.4%) 1: 53 (67.1%) 2: 12 (15.2%) 3: 1 (1.3%) Unknown: 4 (5.1%)	Clear cell: 68 (86.1%) Papillary: 6 (7.6%) Chromophobe: 1 (1.3%) Translocation: 1 (1.3%) Unclassified: 1 (1.3%) Not documented: 2 (2.5%) [5 (6.3%)]	Lung: 42 (53.3%) Lymph node: 20 (25.3%) Bone: 20 (25.3%)
Kwok et al. (2023) [21]	Male: 57 (80.3%) Female: 14 (19.7%)	64 (31–84)	Not reported	Clear cell: 60 (84.5%) Papillary: 3 (4.2%) Chromophobe: 2 (2.8%) Other: 6 (8.5%)	Lung: 35 (49.3%) Lymph node: 20 (28.2%) Liver: 8 (11.3%) Central nervous system: 1 (1.4%) Bone: 25 (35.2%) Muscle: 2 (2.8%) Local tumour bed: 5 (7.0%) Contralateral kidney: 2 (2.8%) Others: 11 (15.5%)
Pal et al. (2022)* [19]	Male: 133 (77.0%); 129 (75.0%) Female: 39 (23.0%); 42 (25.0%)	61 (55–67), 62 (55–68)	0: 128 (74.0%); 124 (73.0%) ≥1: 44 (26.0%): 44 (26.0%) Unknown: 0 (0%); 3 (1.8%)	Not reported	Bone: 59 (34); 64 (37.0%) Brain: 8 (4.7%); 9 (5.3%) Liver: 42 (24.0%); 43 (25.0%) Lung: 114 (66.0%); 124 (73.0%) Lymph node: 97 (56.0%); 99 (58.0%) Adrenal gland: 41 (24.0%); 25 (15.0%) Other: 104 (60.0%); 102 (60.0%)
Buti et al. (2024) [25]	Male: 27 (82.0%)	60 (38–77) Female: 6 (18.0%)	0: 14 (42.0%) 1: 13 (39.0%) 2: 6 (18.0%)	Clear cell: 21 (64.0%) Papillary: 6 (18.0%) Chromophobe: 1 (3.0%) Unclassified: 1 (3.0%) [6 (18.0%)]	Lung: 25 (76.0%) Lymph node: 21 (64.0%) Bone: 18 (55.0%) Liver: 13 (39.0%) Brain: 2 (6.1%) Adrenal gland: 3 (9.1%) Pancreas: 3 (9.1%) Other: 11 (33.0%)
Gavira et al. (2025) [24]	Male: 106 (79.7%) Female: 27 (20.3%)	61 (22–87)	0–1: 72 (54.1%) 2: 36 (27.1%) 3: 11 (8.3%) Unknown: 14 (10.5%)	Clear cell: 109 (82.0%) Papillary: 15 (11.3%) Chromophobe: 3 (2.3%) Others: 6 (4.5%) [22 (16.5%)]	Lung: 91 (68.4%) Lymph node: 95 (71.4%) Liver: 50 (37.6%) Brain: 21 (15.8%) Adrenal gland: 30 (22.6%) Bone: 73 (54.9%) Contralateral kidney: 12 (9.0%) Peritoneum: 31 (23.3%) Pleura: 16 (12.0%) Pancreas: 14 (10.5%)
Lee et al. (2025) [26]	Male: 65 (78.3%) Female: 18 (21.7%)	60 (27–82)	1: 54 (65.1%) 2: 27 (32.1%) Unknown: 2 (2.4%)	Clear cell: 75 (90.4%) Papillary: 6 (7.2%) Unclassified: 2 (2.4%)	Lung: 71 (85.5%) Bone: 38 (45.8%) Liver: 23 (27.7%) Brain: 7 (8.4%)

Note: *All patients’ characteristics from the work by Pak et al. are separately reported for the population receiving lenvatinib at 14 mg dose and the one receiving 18 mg dose, respectively; ECOG PS, Eastern Cooperative Oncology Group Performance Status.

### 3.4 Outcomes

Median OS data showed a marked heterogeneity between the studies. The lowest mOS values were reported in the works by Lee et al. and Kwok et al. (7.5 and 9.0 months, respectively) [21,26], while the longest mOS was observed in Ged et al.’s study (24.5 months), although with significant variability across IMDC groups (data not reported) [20]. In the case series by Hamieh et al., median values for survival outcomes were not reported; OS ranged from 4.0 months to 11.0 months [18]. The remaining four studies presented fewer discrepancies, with mOS ranging from 11.2 to 18.0 months [19,22,23,25].

Median PFS values showed more homogeneity among all studies (between 5.3 and 6.7 months), except for the works by Ged et al. and Pal et al., which reported a mPFS of 12.0 months (significant variability between IMDC groups, data not reported) [20] and 12.9 months (in the population receiving lenvatinib 18 mg) [19], respectively. Two studies did not evaluate mPFS but considered mTTF [21,24]. However, mTTF values did not significantly differ from mPFS values registered in the other studies (5.5 and 6.2 months).

ORR varied across the studies, from the lowest rate of 14.0% [24] to the highest of 55.7% [22]. In the study by Hamieh et al., ORR was not reported [18].

The safety profile of the lenvatinib and everolimus combination was not consistently reported across all studies; only five studies provided data on grade ≥ 3 AEs. Specifically, Pal et al. reported 83 (92.2%) such events in the cohort receiving lenvatinib 14 mg, with diarrhoea being the most frequent [19]; Kwok et al. reported 49 (69.0%) events, also with diarrhoea as the most common [21]; Wiele et al. documented 26 (61.9%) events, with proteinuria being the most frequently observed [23]; Lee et al. described 52 (62.5%) events, also with proteinuria and diarrhoea as the most common [26]; and Buti et al. reported 10 (30.0%) events, primarily hypertension and skin toxicity [25] Overall, the most frequently reported grade ≥ 3 adverse events were diarrhoea and proteinuria.

AEs led to dose reductions of lenvatinib, everolimus, or both, with varying frequencies across the included studies. Lenvatinib dose reductions were reported in 36.7% to 68.0% of patients, while everolimus dose reductions occurred in 11.4% to 18.0% of cases. AE-related discontinuation of the combination therapy ranged from 7.3% to 66.0% across studies. Notably, in the study by Buti et al., no treatment discontinuations due to AEs were reported [18,19,21–25].

All survival outcome results are summarized in Table 1.

All safety profile results are summarized in Table 3.

**Table 3:** Treatment-related adverse events

Study reference, (year)	Adverse events ≥ G3, n (%/range)	Most frequent adverse events (≥G3), n (%)	Adverse event that led to dose reduction, n (%)	Adverse event that led to treatment interruption, n (%)
Hamieh et al. (2020) [18]	Not reported	Not reported	Not reported	2 (28.5%)
Ged et al. (2020) [20]	Not reported	Not reported	Not reported	Not reported
Wiele et al. (2021) [23]	26	Proteinuria: 10 (18.2%); Diarrhoea: 5 (9.1%); Fatigue: 5 (9.1%); Hypertension: 2 (3.6%); Hand and foot syndrome: 2 (3.6%);Decreased appetite: 1 (1.8%); Decreased weight: 1 (1.8%)	Lenvatinib: 28 (50.9%); Everolimus: 7 (16.7%)	4 (7.3%)
Vogelzang et al. (2021) [22]	Not reported	Not reported	Lenvatinib: 29 (36.7%); Everolimus: 9 (11.4%)	21 (32.8%)
Kwok et al. (2023) [21]	49 (69%)	Diarrhoea/colitis: 7 (9.6%); Liver toxicity: 2 (2.8%); Skin toxicity: 4 (5.6%); Kidney damage: 3 (4.2%); Lung toxicity: 3 (4.2%); Mucositis: 5 (7.0%); Musculoskeletal toxicity: 1 (1.4%); Other: 24 (33.8%)	Both drugs: 31 (44.3%)	15 (21.0%)
Pal et al. (2022)* [19]	83 (77–89), 80 (73–86)	Diarrhoea: 27 (16.0%), 29 (17.0%); Hypertension: 18 (10.0%), 25 (15.0%); Proteinuria: 13 (7.5%), 16 (9.5%); Hypertriglyceridemia: 15 (8.7%), 16 (9.5%)	Both drugs: 16 (9.2%), 23 (14.0%); Lenvatinib: 114 (66.0%), 114 (68.0%); Everolimus: 29 (17.0%), 30 (18.0%)	Both drugs: 100 (58.0%), 111 (66.0%) Lenvatinib: 105 (61.0%), 117 (70.0%) Everolimus: 126 (73.0%), 137 (82.0%)
Buti et al. (2024) [25]	10 (30.0%)	Diarrhoea: 1 (3.0%); Fatigue: 1 (3.0%); Stomatitis: 1 (3.0%); Nausea: 1 (3.0%); Hypertension: 2 (6.0%); Skin toxicity: 2 (6.0%); Interstitial pneumonia: 1 (3.0%); Dyspnea: 1 (3.0%)	Both drugs: 12 (36.0%)	0 (0%)
Gavira et al. (2025) [24]	Not reported	Not reported	Everolimus: 8 (17.7%)	Both drugs: 6 (13.3%)
Lee et al. (2025) [26]	52 (62.5%)	Proteinuria: 19 (22.9%) Diarrhoea: 9 (10.8%) Hypertension: 5 (6.0%)	Lenvatinib: 46 (55.4%) Everolimus: 15 (18.1%)	Lenvatinib: 22 (26.5%) Everolimus: 22 (26.5%)

Note: *All the adverse events from the work by Pal et al. are separately reported for the population receiving Lenvatinib at 14 mg dose and the one receiving 18 mg dose, respectively; G3, grade 3.

## 4 Discussion

The introduction of first-line ICIs combinations in mRCC has fundamentally changed treatment paradigms and highlighted the complexity of defining optimal sequencing in the second and subsequent-line settings. This systematic review, conducted in accordance with PRISMA guidelines, synthesizes the available evidence on the efficacy and safety of the lenvatinib–everolimus combination after ICI-based regimens and identifies the main limitations of the included studies.

Robert Motzer et al. evaluated the efficacy of lenvatinib and everolimus combination over everolimus monotherapy or lenvatinib monotherapy, after progression on a VEGFR-TKI treatment. This trial (Study 205) demonstrated a mPFS benefit of the lenvatinib and everolimus combination and of lenvatinib monotherapy over everolimus monotherapy, leading to the FDA and EMA approval of the combination in 2016 [11]. Since then, little real-world data have been collected to describe the effectiveness of this combination after ICI-based treatment.

Preclinical data from murine models have demonstrated that lenvatinib induces tumour immune microenvironment modifications (e.g., reduction of tumour-associated macrophages (TAMs) and increase of activated CD8+ T cells secreting interferon (IFN)-γ+ and granzyme B). These immunomodulations promote antineoplastic activity through the type-I and type-II IFN signalling pathway, therefore increasing antitumour activity exerted by anti-PD-1 ICIs [27].

The concept of a persistent immunologic effect after ICI discontinuation is clinically relevant. Results of the phase III trial CONTACT-03 showed that, after progression on a previous ICIs-containing regimen, adding atezolizumab to cabozantinib did not improve clinical outcomes. These negative results may depend on the persistent activity exerted by previous immunotherapy in control arm (receiving cabozantinib monotherapy) [28], furtherly supporting the use of a lenvatinib-based combination after ICIs discontinuation. Similar results were reported in the TiNivo-2 trial, with no benefits in terms of mPFS for the population receiving tivozanib and nivolumab, compared to the patients receiving tivozanib monotherapy [29].

Across the studies included in our review, the combination demonstrated consistent activity after ICI failure, both in the second- and further-lines settings, with mPFS ranging from 6.1 to 12.9 months and mOS up to 18.0 months—comparable to outcomes reported for cabozantinib or tivozanib in similar post-ICI settings [28,29].

Safety analyses confirmed a manageable profile across all IMDC risk groups with no unexpected toxicities, and treatment discontinuation rates appeared lower than in Study 205. In addition, our findings suggest that survival outcomes following lenvatinib dose reduction described in this review are consistent with expectations in clinical practice. The most frequently reported ≥G3 AEs were diarrhoea, proteinuria and hypertension though these occurred in a relatively small proportion of patients. In contrast, lower-grade AEs were more common and persisted for longer durations. These chronic toxicities may have a more significant impact on patients’ quality of life than the acute, higher-grade AEs; however, these specific quality of life data were not systematically collected in the included studies. Moreover, the work by Pal et al. showed worse ORR and mPFS with similar AE-related dose reduction rates in the lenvatinib 14 mg quaque die (QD) arm compared to Study 205, therefore supporting the Lenvatinib 18 mg QD as the most appropriate initial dose [12,19]. On the other hand, in the work by Lee et al., almost 90% of the study population received lenvatinib at an initial reduced dose, with an alternating 20 and 10 mg QD as the most common dosing pattern. Despite such a different posology, ORR and survival outcomes were comparable to the ones reported in the other studies included in our review [26]. Therefore, defining the most appropriate dosage for lenvatinib and everolimus in the present setting still represents a challenging topic.

The present systematic review offers an updated and comprehensive summary of the clinical evidence supporting the use of the lenvatinib and everolimus combination in a more contemporary therapeutic context and, to our knowledge, no other published reviews have explored such a specific topic.

Current international guidelines (ESMO 2024, NCCN 2025) list multiple agents for post-ICI treatment, including lenvatinib–everolimus, but without a clear hierarchy, reflecting heterogeneity in available data and the predominance of indirect evidence. European guidelines recommend VEGFR-targeted sequencing, although this is largely based on pre-immunotherapy-era trials and control arms of post-ICI studies [28,29].

Importantly, there are no ongoing phase III trials directly comparing lenvatinib–everolimus to other regimens in the post-ICI setting, likely due to the difficulty of selecting the most appropriate control arm. A phase II trial comparing the combination with cabozantinib monotherapy as second-line treatment after progression on an ICI-containing regimen is ongoing (NCT05012371) [30] and may better define the role of lenvatinib and everolimus combination in therapeutic sequencing.

Moreover, results from the phase II trial LITESPARK-003 described promising activity from the combination of cabozantinib with a HIF-2α inhibitor, belzutifan [31]. Following these data, two randomized phase III trials have been initiated: LITESPARK-011 will assess efficacy and safety of belzutifan and lenvatinib combination vs cabozantinib, after progression on an ICIs-based regimen; PEAK-1 will evaluate efficacy and safety of casdatifan (a highly specific HIF-2α inhibitor) and cabozantinib combination vs cabozantinib, also after prior ICIs-based therapy. Results from these studies are still awaited [32,33].

Notably, the biological rationale for combining an HIF-2α inhibitor with a multi-targeted VEGFR-TKI may also support the inclusion of an mTOR inhibitor, which indirectly downregulates the HIF pathway.

Our review has relevant limitations that should be acknowledged. The included studies differed in several key aspects, including study population size, distribution of prognostic groups, treatment lines, and survival outcomes. In the study conducted by Ged et al., only 4 of the 59 enrolled patients received the lenvatinib plus everolimus combination. Although this study reported the longest median overall survival (24.5 months) and median PFS (12.0 months) among the available data, the small number of patients treated with this regimen could potentially inflate survival estimates [20]. Moreover, the completeness of the review process was inherently limited by the absence of reported methodological details (e.g., risk of bias and certainty assessments) within the included studies.

Despite the majority of the studies included in our analysis being retrospective in design, we also incorporated a randomized, prospective phase II trial, which may contribute more robust evidence to support the findings of this review [19].

## 5 Conclusions

The current review might support the use of the lenvatinib and everolimus combination in later lines of therapy for patients with mRCC, even in the era of ICIs. Real-world data from retrospective studies and a prospective randomized phase II trial have confirmed the combination’s effectiveness following immunotherapy failure, without showing any unexpected changes to its well-known toxicity profile.

However, larger prospective trials are still necessary to provide more robust evidence and fully inform clinical decision-making.

## Supplementary Materials



## Data Availability

Data sharing not applicable to this article as no datasets were generated or analysed during the current study.

## Supplementary Materials

The supplementary material is available online at https://www.techscience.com/doi/10.32604/or.2025.070523/s1.
