# Supplementary material for: Effectiveness and Safety of Lenvatinib and Everolimus after Immune Checkpoint Inhibitors in Metastatic Renal Cell Cancer: A Systematic Review
Source: Oncol Res. 2025 Dec 30;34(1):3. doi: 10.32604/or.2025.070523 (PMC12774557; doi:10.32604/or.2025.070523)
Supplement: Supplementary file 1 [file OncolRes-34-70523-s001.docx]

**PICO**

**Question:**

What is the effectiveness exerted by the lenvantinib and everolimus combination in metastatic renal cell carcinoma (mRCC) after progression on an immune-checkpoint inhibitor (ICI)-based regimen?



- The primary objective of this review is to determine the effectiveness of lenvatinib and everolimus combination.
- The secondary objective of this review is to determine safety and toxicity profile of lenvatinib and everolimus combination.

**P (Population/patient):**

- Inclusion Criteria:
  - Patients affected by mRCC
  - Lenvatinib and everolimus combination treatment
  - Previous treatment with ICI-based regimen
  - Prospective and retrospective studies
  - Studies published between 2020 and 2025
- Exclusion criteria
  - Meta-analysis
  - Systematic review
  - Case-control studies
  - Editorials
  - Commentaries

**I (intervention):**

Lenvatinib and everolimus combination as second or further lines treatment.

**C (Comparison):**

No other treatment regimen for comparison.

**O (Outcome):**

- Primary outcomes:
  - Median overall survival
  - Median progressive free survival
  - Objective response rate
- Secondary outcome:
  - Treatment related adverse events

**GRADE**

**High certainty level**

We are **very confident** that the true effect lies close to that of the estimate of the effect.

- Evidence from well-conducted randomized studies without major limitations.
- Further research is unlikely to change the estimate.

**Moderate certainty level**

We are **moderately confident** in the effect estimate: the true effect is likely to be close to the estimate, but there is a possibility it is substantially different.

- Some methodological limitations, inconsistency, or imprecision.
- Further studies may have a significant impact on our confidence and the estimate.

**Low certainty level**

Our confidence in the effect estimate is limited: the true effect may be substantially different from the estimate.

- Evidence from observational studies or from RCTs with serious limitations.
- It is likely that further research will change the estimate.

**Very low certainty level**

We have **very little confidence** in the effect estimate: the true effect is likely to be substantially different from the estimate.

- Very indirect, imprecise, or substantially biased evidence.
- The estimate is very uncertain.
